# Supplementary material for: MTG8 interacts with LHX6 to specify cortical interneuron subtype identity
Source: Nat Commun. 2022 Sep 5;13:5217. doi: 10.1038/s41467-022-32898-6 (PMC9445035; doi:10.1038/s41467-022-32898-6)
Supplement: Supplementary file 1 — Supplementary Information [file 41467_2022_32898_MOESM1_ESM.pdf]

Supplementary information

MTG8 interacts with LHX6 to specify cortical interneuron subtype identity

Zeinab Asgarian[1]*, Marcio Guiomar Oliveira[1]*, Agata Stryjewska[1]*, Ioannis Maragkos[2], Anna Noren Rubin[1], Lorenza Magno[1], Vassilis Pachnis[3], Mohammadmersad Ghorbani[4,5], Scott Wayne Hiebert[6], Myrto Denaxa[2] and Nicoletta Kessaris[1]

[1] *Wolfson Institute for Biomedical Research, University College London, Gower Street, London WC1E 6BT, UK*

[2] *Biomedical Sciences Research Center "Alexander Fleming", Vari, Greece*

[3] *The Francis Crick Institute, London, UK.*

[4] *Centre for Cancer Immunology, Faculty of Medicine, University of Southampton, Southampton General Hospital, Southampton, UK*

[5] *Department of Human Genetics, Sidra Medicine, Doha, Qatar*

[6] *Department of Biochemistry, Vanderbilt University School of Medicine, Nashville, USA*

* Equally contributing authors

Correspondence to: n.kessaris@ucl.ac.uk

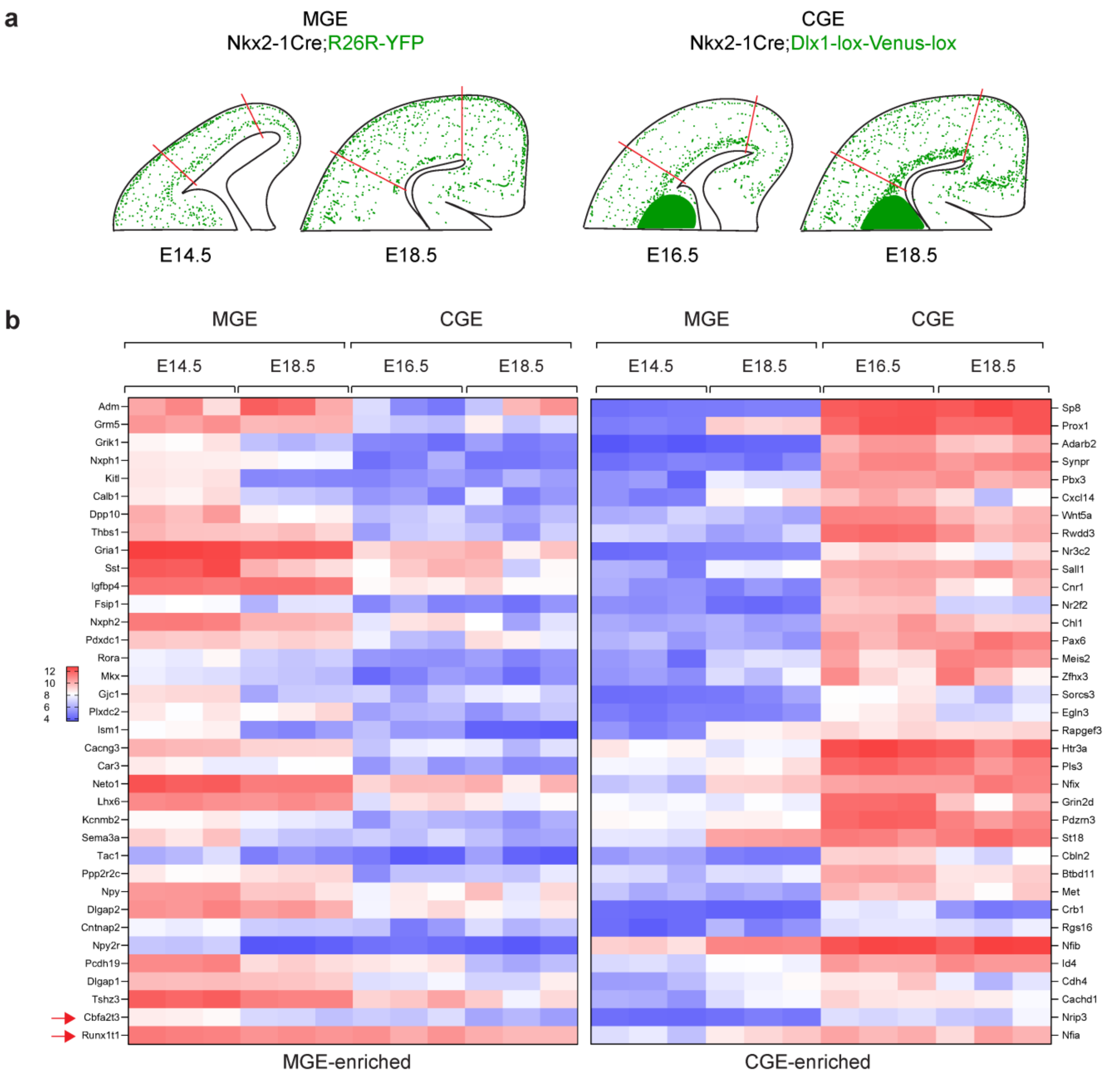

Supplementary Fig.1 | Transcriptomic profiling of MGE- and CGE-derived cells migrating within the developing neocortex at embryonic stages

a, Experimental design for transcriptomic profiling of migrating MGE and CGE cells. Embryonic brains from mice expressing YFP/VENUS in MGE-derived cells (Nkx2-1-Cre;R26R-YFP) or CGE-derived migrating interneurons (Nkx2-1-Cre;Dlx1-lox-Venus-lox) were used to isolate fluorescent cells from the developing neocortex at different developmental stages (E14.5 and E18.5 for the MGE and E16.5 and E18.5 for the CGE). Purified cells were processed for transcriptomic analysis using Microarrays.

b, Heat maps showing some of the top hits among the MGE- and CGE-enriched genes. *Mtg8* (*Runx1t1*) and *Mtg16* (*Cbfa2t3*) (arrows) were identified as being enriched in MGE cells.

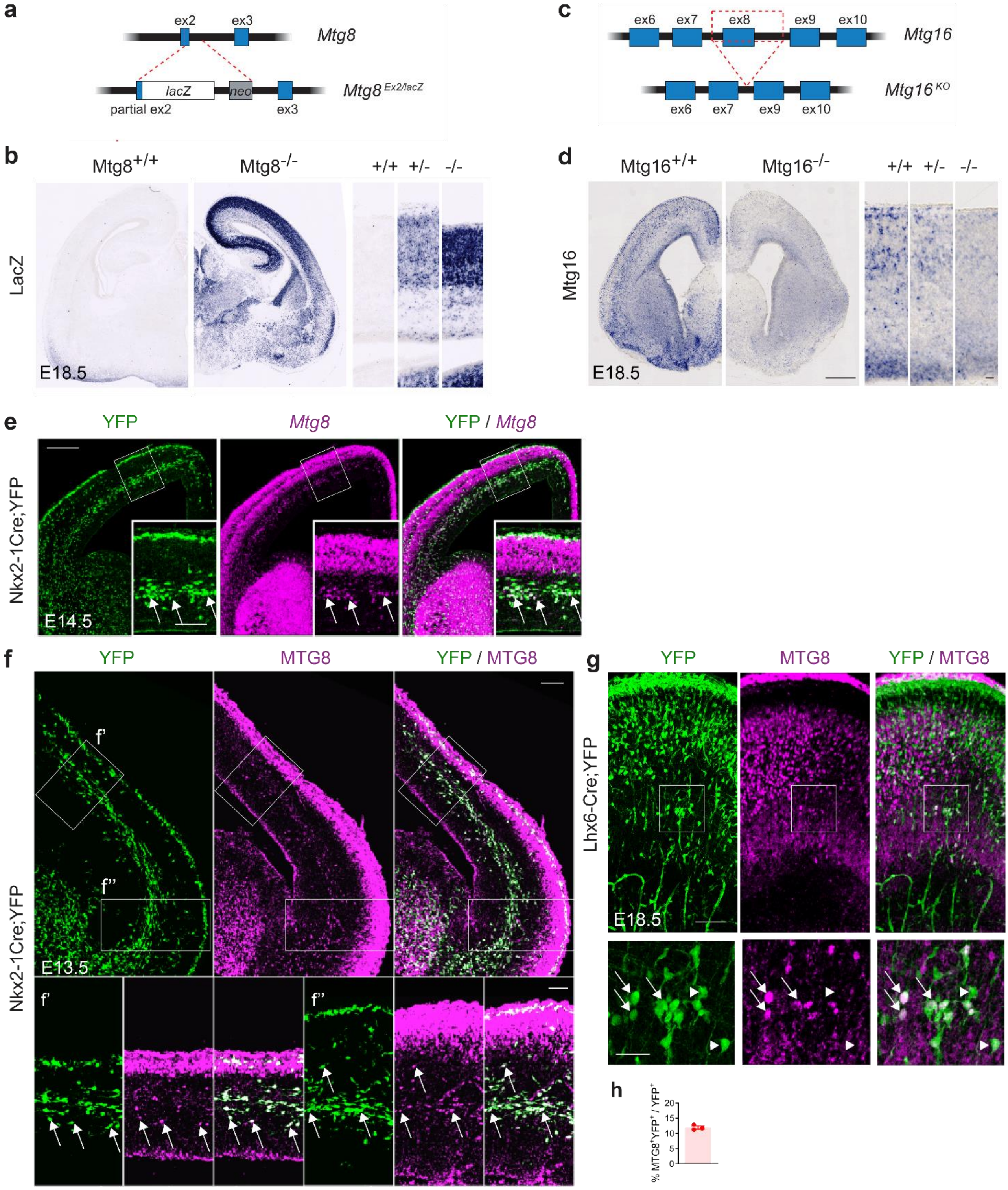

Supplementary Fig.2

a-d, Germline loss-of-function mice used in this study

a, *Mtg8* mice include a LacZ reporter gene inserted into exon 2.

b, *LacZ* expression can be detected in heterozygote and homozygote animals using RNA ISH.

c, *Mtg16* mice used in this study.

d, RNA ISH for *Mtg16* shows reduction and loss of transcript expression in heterozygote and homozygote animals, respectively.
Scale bars: b,d 500 µm and higher magnifications 30 µm.

e, Expression of *Mtg8* in the cortex in migrating cortical interneurons and in the cortical plate.
FISH assay for *Mtg8* followed by immunohistochemistry for YFP in Nkx2-1Cre;YFP embryos shows extensive co-localization between *Mtg8* and YFP in migrating MGE-derived interneurons in the developing cortex at E14.5 (arrows).
Scale bar: 250 µm and higher magnifications 100 µm.

f-h, Immunohistochemistry for MTG8 and YFP in MGE lineage tracing mice
f, MTG8 is expressed in migrating MGE interneurons in the embryonic cortex at E13.5
g-h, Expression of MTG8 in cortical MGE interneurons at E18.5 (arrows) and quantification. n = 3 brains. Graph shows mean ± SEM. Source data are provided as a Source Data file.

Scale bars: f, 100 µm and higher magnifications 25 µm; g, 75 µm and higher magnification 25 µm.

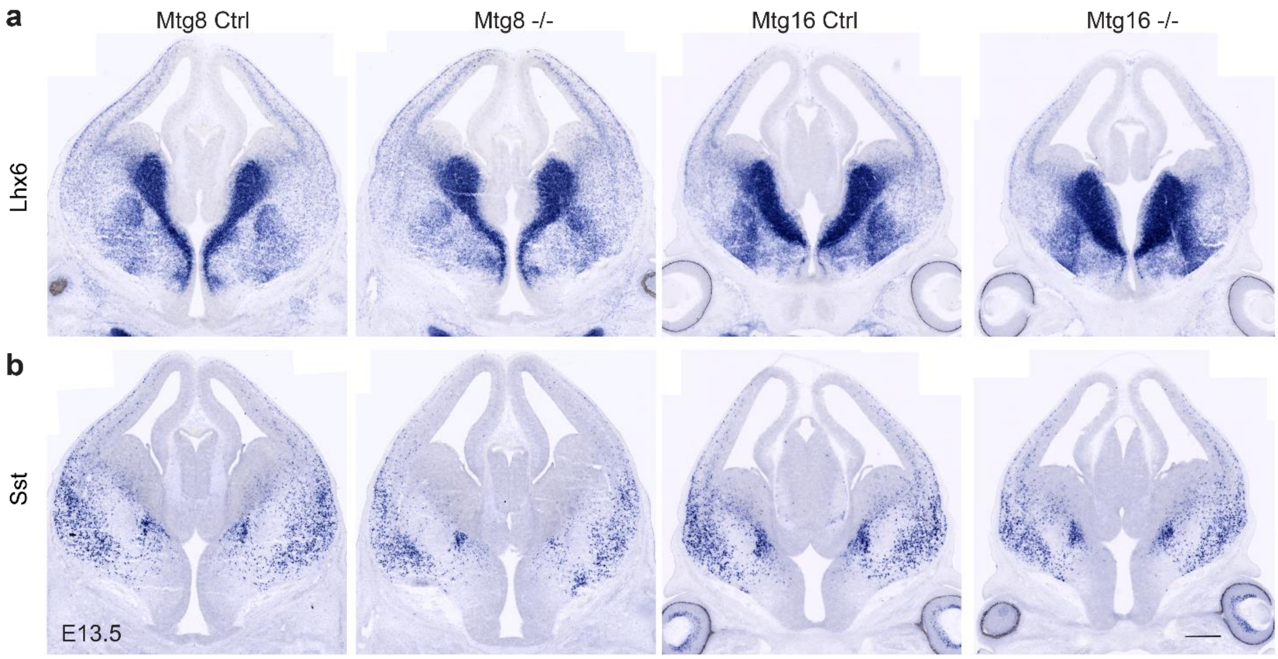

Supplementary Fig.3 | Distribution of *Lhx6* and *Sst* transcripts in the embryonic telencephalon in control and mutant embryos

a-b, Low magnification of E13.5 telencephalon images shown in Figure 1a, b indicating expression of *Lhx6* and *Sst*

a, Distribution of *Lhx6* in control (Ctrl), *Mtg8*$^{-/-}$ and *Mtg16*$^{-/-}$ embryos. The MGE mantle is demarcated by *Lhx6.*

b, Distribution of *Sst* in control (Ctrl), *Mtg8*$^{-/-}$ and *Mtg16*$^{-/-}$ embryos

Scale bar 500 µm.

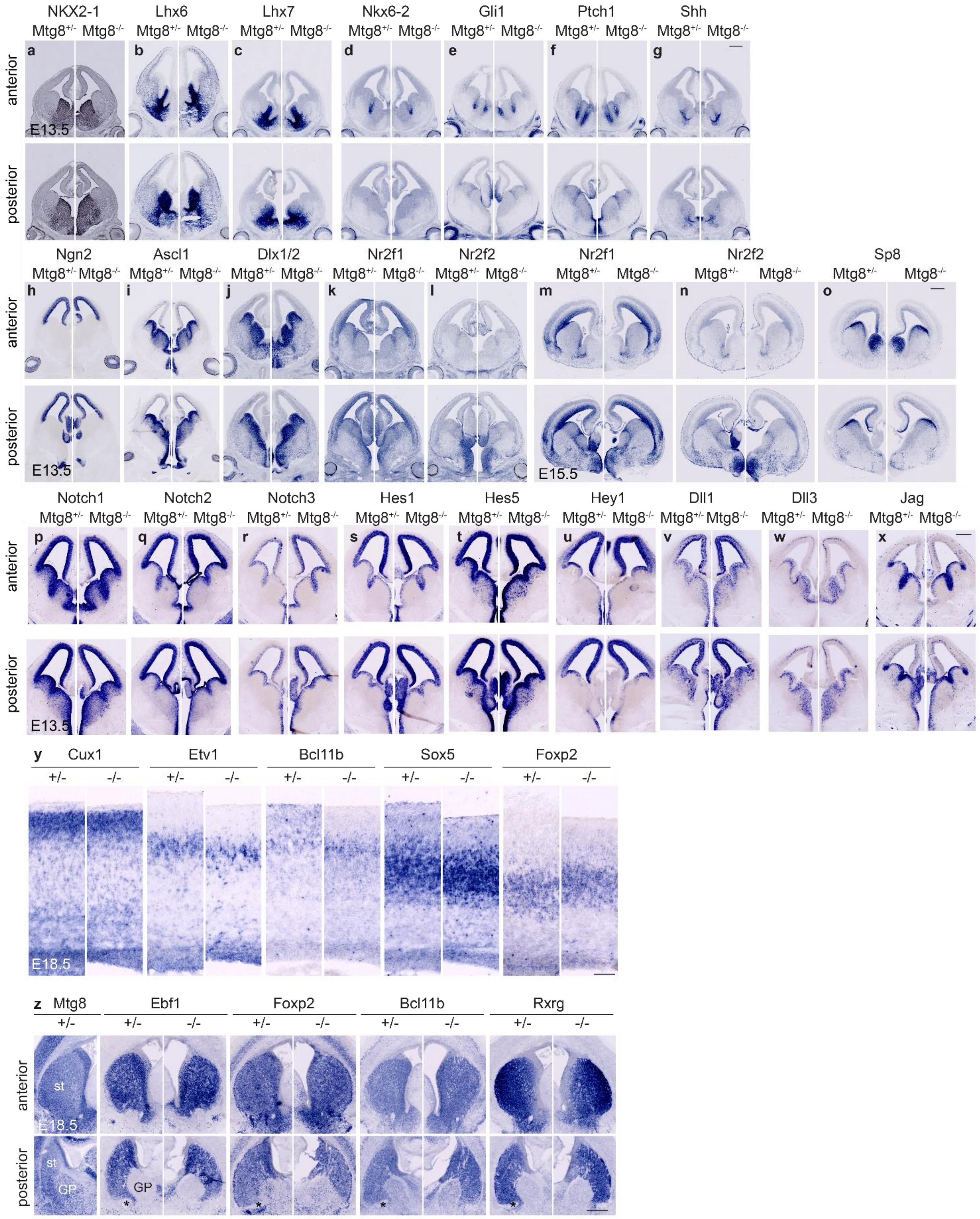

Supplementary Fig.4 | Normal expression of genetic markers in the embryonic telencephalon in the absence of *Mtg8* or *Mtg16*

a-o, Immunohistochemistry (a) and RNA ISH (b-o) in the telencephalon showing expression of several patterning and other genes in control (Ctrl), *Mtg8*$^{-/-}$ and *Mtg16*$^{-/-}$ embryos at E13.5 and E15.5. The MGE is demarcated by NKX2-1. Scale bars: a-l, 500 µm; m-o, 500 µm.

p-x, RNA ISH in the telencephalon showing expression of *Notch-Delta* pathway genes in control (Ctrl), *Mtg8*$^{-/-}$ and *Mtg16*$^{-/-}$ embryos at E13.5. Scale bar 500 µm.

y, RNA ISH in the cortex showing expression of layer-specific genes in control (Ctrl), *Mtg8*$^{-/-}$ and *Mtg16*$^{-/-}$ embryos at E18.5. Scale bar 75 µm.

z, RNA ISH showing expression of basal ganglia genes in control (Ctrl), *Mtg8*$^{-/-}$ and *Mtg16*$^{-/-}$ embryos at E18.5. Asterisks indicate the anterior commissure in control embryos. St, striatum; GP, globus pallidus. Scale bar 500 µm.

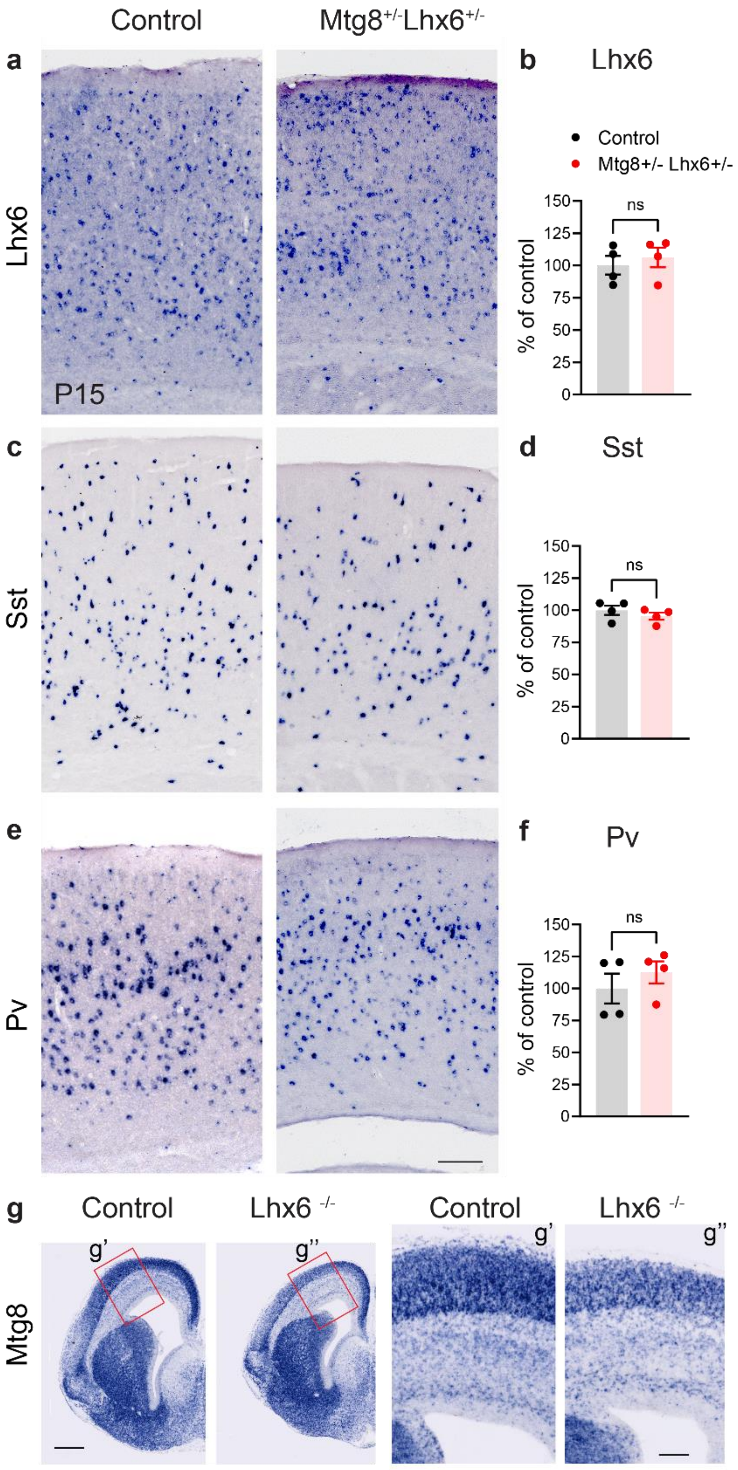

Supplementary Fig.5 | Lack of epistatic interactions between *Lhx6* and *Mtg8*

a-f, Normal numbers of *Lhx6* (a-b), *Sst* (c-d) and *Pv* (e-f) cells in the cortex of double heterozygote ($Mtg8^{+/-}Lhx6^{+/-}$) mice compared to controls at P15. n = 4 brains per genotype. b, d, Two-tailed unpaired *t* tests. *f*, Two-tailed Mann-Whitney U test.

g, E18.5 brains from control and *Lhx6* mutant embryos. Normal expression of *Mtg8* in the absence of Lhx6 in the telencephalon (left two images) and in the cortex (right two images).

All graphs show mean ± SEM. Source data are provided as a Source Data file.

Scale bars: a, c, e: 130 µm; g, left images 350 µm, right images 85 µm.

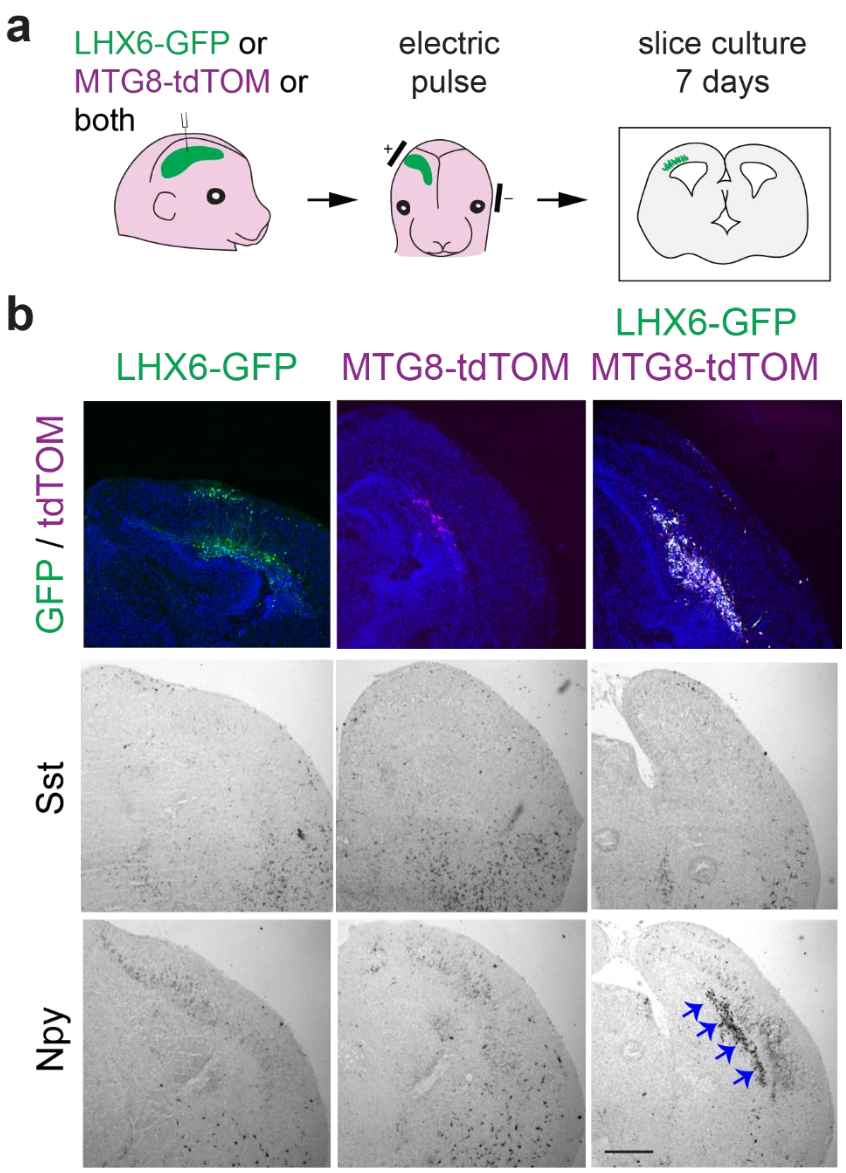

Supplementary Fig.6 | Upregulation of *Npy* upon ectopic expression of MTG8 and LHX6 in the developing neocortex

a, Experimental scheme for ectopic expression of LHX6-GFP, or MTG8-tdTOM, or both, in the embryonic cortex via whole head electroporation at E14.5-E15.5, followed by slice culture of coronal sections from electroporated brains (b).

b, Expression of *Sst,* and *Npy* in the developing cortex upon ectopic expression of MTG8 and/or LHX6. *Npy* is upregulated only in the presence of both, MTG8 and LHX6.

Scale bar 300 µm.

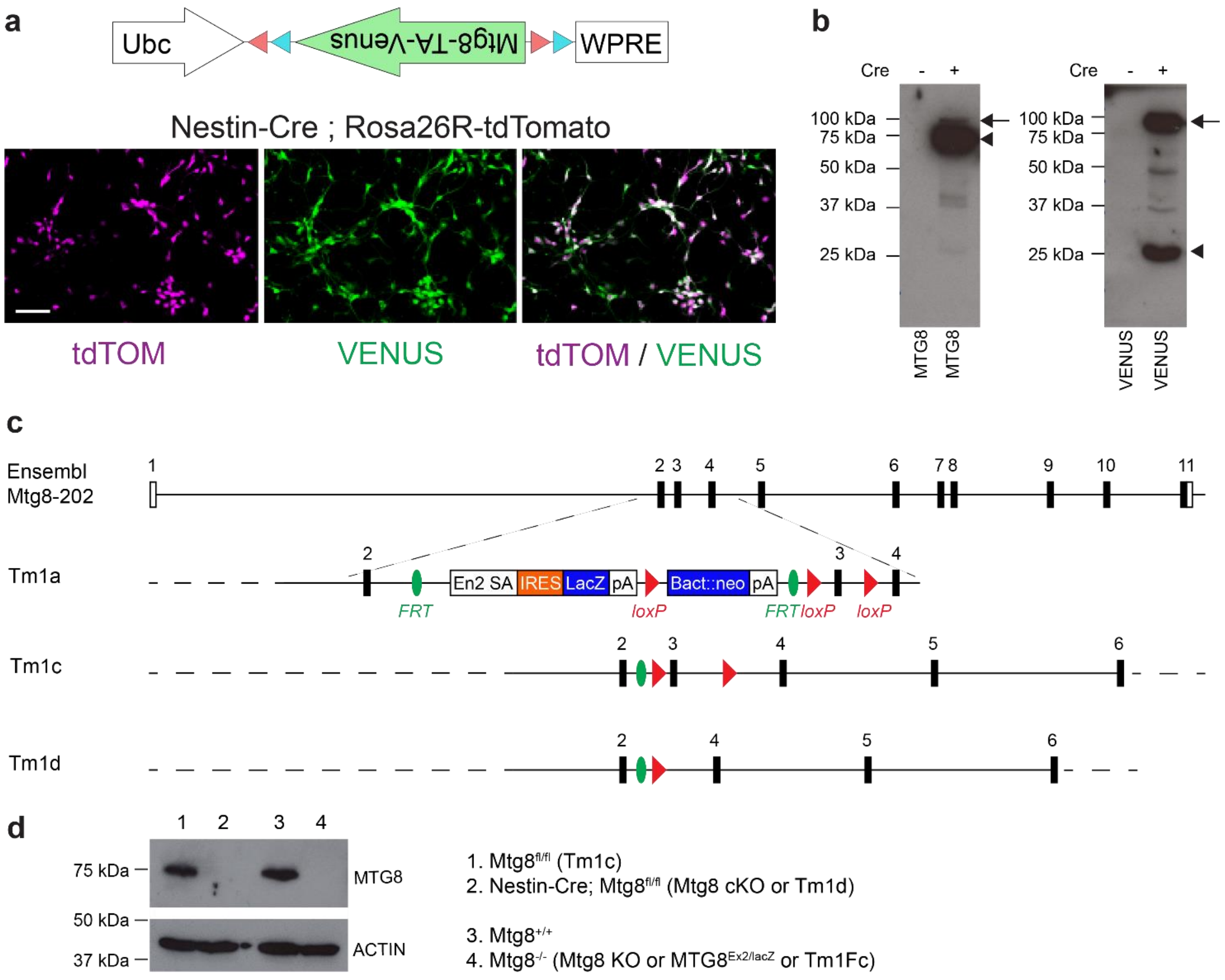

Supplementary Fig. 7

a-b, Validation of the MTG8-TA-VENUS-expressing Lentivirus

a, Schematic of the lentiviral construct used to overexpress *Mtg8* in MGE-derived cells. The lentiviral construct is double floxed, allowing restricted expression of the cassette in cells carrying Cre. The T2A sequence mediates the multi-protein self-cleavage to separate MTG8 and VENUS proteins. Immunohistochemistry detecting VENUS and TdTomato in MGE cells isolated from Nestin-Cre;Tdtom mice and transduced with Mtg8-TA-Venus virus. Scale bar 50 µm.

b, Western blot analysis of protein extracts prepared from Cos-7 cells that had been co-transfected with the Mtg8-TA-Venus plasmid in the presence or absence of a Cre-expressing plasmid. Cleavage between VENUS and MTG8 can be seen upon detection of MTG8 or VENUS proteins. Arrowheads indicate cleaved proteins. Arrows point to un-cleaved polypeptides.

c-d, Generation and validation of a Cre conditional *Mtg8* mouse

c, Schematic showing the modification of the *Mtg8* locus. Tm1a represents the knock-out-first allele. Tm1c represents the conditional allele and tm1d represents the deleted allele following Cre recombination.

d, Western blot showing loss of MTG8 protein in embryonic brains from Nestin-Cre;Mtg8$^{fl/fl}$ mice and the *Mtg8* mutant mice (Mtg8$^{Ex2/LacZ}$).

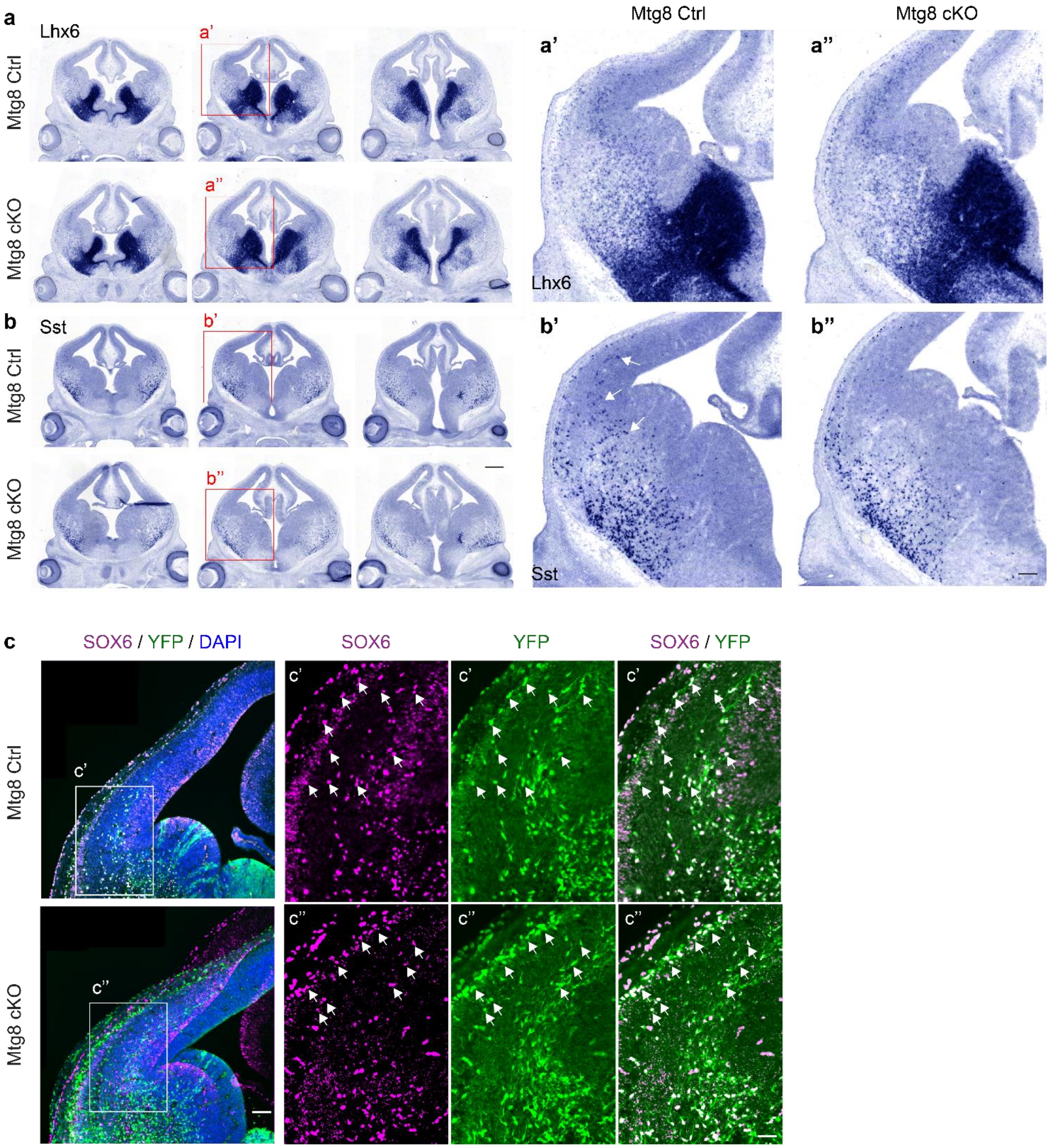

Supplementary Fig.8 | Distribution of *Lhx6* and *Sst* transcripts as well as SOX6 protein in the embryonic telencephalon of control and *Mtg8* conditional mutant embryos at E13.5

a, Distribution of *Lhx6* in control (Ctrl) (Mtg8$^{fl/fl}$) and *Mtg8* cKO (Nkx2-1-Cre;Mtg8$^{fl/fl}$) embryos. The red box areas in a are shown at higher magnification in a' and a''.

b, Distribution of *Sst* in control (Ctrl) (Mtg8$^{fl/fl}$) and *Mtg8* cKO (Nkx2-1-Cre;Mtg8$^{fl/fl}$) embryos. The red box areas in b are shown at higher magnification in b' and b''. White arrows in b' point to *Sst* cells in the SVZ stream that are reduced/missing in the absence of MTG8 (b'').

c, Expression of SOX6 in migrating MGE cells co-expressing YFP in *Mtg8* control (Ctrl) (Nkx2-1-Cre;Mtg8$^{+/+}$;R26R-YFP) and *Mtg8* cKO (Nkx2-1-Cre;Mtg8$^{fl/fl}$;R26R-YFP) embryos. White arrows point to migrating SOX6/YFP cells in the MZ and SVZ streams.

Scale bars: a,b 500 μm; a',a'',b',b'' 140 μm; c, 125 μm; c',c'' 65 μm.

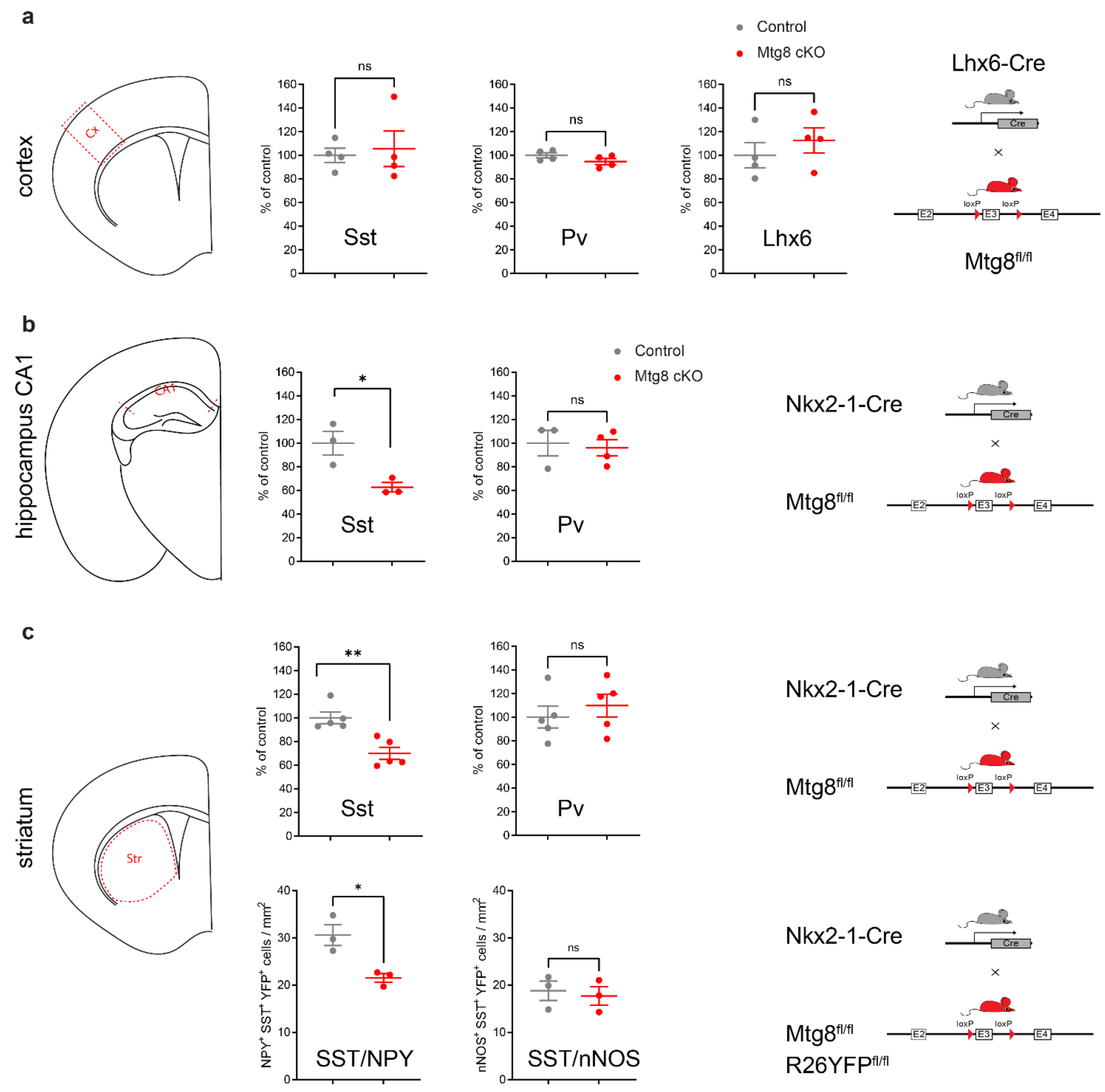

Supplementary Fig.9 | Analysis of *Mtg8* cKO mice at P40 upon late (a) or early (b) deletion of *Mtg8* in the MGE lineage

a, Late deletion of *Mtg8* in migrating MGE interneurons using Lhx6-Cre.

Normal numbers of *Sst, Pv* and *Lhx6* cells in the somatosensory cortex.

n = 4 brains per genotype. Mean ± SEM. Two-tailed unpaired *t* tests.

b, c, Early deletion of *Mtg8* in migrating MGE cells using Nkx2-1-Cre

b, Reduced *Sst* but normal *Pv* interneuron numbers in the hippocampal CA1 area in *Mtg8* cKO animals compared to controls. n = 3 brains per genotype. Mean ± SEM. Two-tailed unpaired *t* tests. *p = 0.0263.

c, Reduced *Sst* but normal *Pv* interneuron numbers in the striatum of *Mtg8* cKO animals compared to controls. Reduced SST interneurons co-expressing NPY, but normal SST interneurons co-expressing nNOS in the striatum of *Mtg8* cKO animals compared to controls. *Sst* and *Pv*, n = 5 brains per genotype. SST/NPY and SST/nNOS n = 3 brains per genotype. Mean ± SEM. Two-tailed unpaired *t* tests. *p = 0.0192. **p = 0.0027.

Source data are provided as a Source Data file.
