# Supplementary material for: MTG8 interacts with LHX6 to specify cortical interneuron subtype identity
Source: Nat Commun. 2022 Sep 5;13:5217. doi: 10.1038/s41467-022-32898-6 (PMC9445035; doi:10.1038/s41467-022-32898-6)
Supplement: Supplementary file 3 — Description of Additional Supplementary Files [file 41467_2022_32898_MOESM3_ESM.pdf]

**Description of Additional Supplementary Files**

File Name: Supplementary Data 1
Description: Genes significantly enriched (FDR < 0.05) in MGE versus CGE or CGE versus MGE at early stages (E14.5 for MGE and E16.5 for CGE). Negative fold change (FC) values indicate MGE enrichment. Some genes/transcripts are represented by more than one probe on the microarray; in these cases, the probe with the highest FC value is listed. Only probes assigned to a known or predicted gene are shown. Note that MGE-enriched genes include genes expressed in migrating interneurons as well as oligodendrocyte lineage cells. CGE-enriched genes include only genes expressed in migrating interneurons.

File Name: Supplementary Data 2
Description: Differentially expressed genes in migrating MGE-derived cortical interneurons lacking either *Mtg8* or *Lhx6*. Negative FC values indicate decreased expression in mutants compared to controls. Genes with a p-adj value of < 0.05 (adjusted for multiple comparisons using DeSeq2 in R and the two-tailed Wald test) are shown.

File Name: Supplementary Data 3
Description: DNA constructs/plasmids used in this study.
