# Supplementary material for: MTG8 interacts with LHX6 to specify cortical interneuron subtype identity
Source: Nat Commun. 2022 Sep 5;13:5217. doi: 10.1038/s41467-022-32898-6 (PMC9445035; doi:10.1038/s41467-022-32898-6)
Supplement: Supplementary file 7 — Reporting Summary [file 41467_2022_32898_MOESM7_ESM.pdf]

nature portfolio

Corresponding author(s): Nicoletta Kessaris

Last updated by author(s): 13 August 2022

Reporting Summary

Nature Portfolio wishes to improve the reproducibility of the work that we publish. This form provides structure for consistency and transparency in reporting. For further information on Nature Portfolio policies, see our Editorial Policies and the Editorial Policy Checklist.

Statistics

For all statistical analyses, confirm that the following items are present in the figure legend, table legend, main text, or Methods section.

n/a	Confirmed	
☐	☒	The exact sample size (*n*) for each experimental group/condition, given as a discrete number and unit of measurement
☐	☒	A statement on whether measurements were taken from distinct samples or whether the same sample was measured repeatedly
☐	☒	The statistical test(s) used AND whether they are one- or two-sided<br>*Only common tests should be described solely by name; describe more complex techniques in the Methods section.*
☒	☐	A description of all covariates tested
☐	☒	A description of any assumptions or corrections, such as tests of normality and adjustment for multiple comparisons
☐	☒	A full description of the statistical parameters including central tendency (e.g. means) or other basic estimates (e.g. regression coefficient) AND variation (e.g. standard deviation) or associated estimates of uncertainty (e.g. confidence intervals)
☐	☒	For null hypothesis testing, the test statistic (e.g. *F*, *t*, *r*) with confidence intervals, effect sizes, degrees of freedom and *P* value noted<br>*Give P values as exact values whenever suitable.*
☒	☐	For Bayesian analysis, information on the choice of priors and Markov chain Monte Carlo settings
☒	☐	For hierarchical and complex designs, identification of the appropriate level for tests and full reporting of outcomes
☒	☐	Estimates of effect sizes (e.g. Cohen's *d*, Pearson's *r*), indicating how they were calculated

Our web collection on statistics for biologists contains articles on many of the points above.

Software and code

Policy information about availability of computer code

Data collection	No software was used to collect data.
Data analysis	Raw sequence data were adapter and quality trimmed [using fastp v0.20.1, available on https://github.com/OpenGene/fastp], aligned to the genome with STAR [v2.7b available on https://github.com/alexdobin/STAR] and reads per transcript determined with FeatureCounts [v1.6.4, available at http://subread.sourceforge.net/]. Differential expression was performed using DESeq2 in R [https://www.bioconductor.org/].

For manuscripts utilizing custom algorithms or software that are central to the research but not yet described in published literature, software must be made available to editors and reviewers. We strongly encourage code deposition in a community repository (e.g. GitHub). See the Nature Portfolio guidelines for submitting code & software for further information.

Data

Policy information about availability of data

All manuscripts must include a data availability statement. This statement should provide the following information, where applicable:

- Accession codes, unique identifiers, or web links for publicly available datasets
- A description of any restrictions on data availability
- For clinical datasets or third party data, please ensure that the statement adheres to our policy

All sequencing data produced for this study have been deposited to the Gene Expression Omnibus (GEO) database under accession numbers GSE195743 and GSE207506.

Field-specific reporting

Please select the one below that is the best fit for your research. If you are not sure, read the appropriate sections before making your selection.

☒ Life sciences ☐ Behavioural & social sciences ☐ Ecological, evolutionary & environmental sciences

For a reference copy of the document with all sections, see nature.com/documents/nr-reporting-summary-flat.pdf

Life sciences study design

All studies must disclose on these points even when the disclosure is negative.

Sample size	No statistical methods were used to pre-dermine sample sizes. Sample sizes were chosen based on extensive experience with such experiments and following literature standards.
Data exclusions	No data were excluded from the analysis
Replication	A minimum of 3 replicates was performed for each experiment. The exact replication is indicated in Methods.
Randomization	Not relevant for this study
Blinding	Where appropriate, investigators were blind to the genotypes. This included all quantitative work.

Reporting for specific materials, systems and methods

We require information from authors about some types of materials, experimental systems and methods used in many studies. Here, indicate whether each material, system or method listed is relevant to your study. If you are not sure if a list item applies to your research, read the appropriate section before selecting a response.

Materials & experimental systems

n/a	Involved in the study
☐	☒ Antibodies
☐	☒ Eukaryotic cell lines
☒	☐ Palaeontology and archaeology
☐	☒ Animals and other organisms
☒	☐ Human research participants
☒	☐ Clinical data
☒	☐ Dual use research of concern

Methods

n/a	Involved in the study
☒	☐ ChIP-seq
☒	☐ Flow cytometry
☒	☐ MRI-based neuroimaging

Antibodies

Antibodies used

rat anti-GFP IgG2a (1:1000; Nacalai Tesque, Kyoto,Japan, #04404-84)
rabbit anti-GFP (1:5000; Abcam, UK, #ab290)
chicken anti-GFP (1:500; Aves Labs, #GFP-1020);
rabbit anti-β-galactosidase (1:2000, MP Biomedicals, # 0856032)
goat anti-MTG8 (1:200, Santa Cruz Biotechnology, #sc-9737)
rabbit anti-MTG16 (1:300, Abcam, UK, #ab33072)
rabbit anti-PV (1:1000, Chemicon Millipore, #MAB1572)
mouse anti-PV (1:1000, Swant Bellizona Switzerland, #235)
rabbit anti-SST (1:200, Peninsula Labs, #T-4103.0050)
rat anti-SST (1:500, Chemicon Millipore, #MAB354)
rabbit anti-calretinin (1:1000, Swant Bellizona Switzerland, #7697)
mouse anti-CR (1:500, Swant Bellizona Switzerland, #6B3)
rabbit anti-NPY (1:1000, ImmunoStar, #22940)
sheep anti-NPY (1:500; Abcam, UK, # ab6173)
rabbit anti-nNOS (1:1000; Immunostar, #24287)
rabbit anti-SOX6 (1:500, Abcam #ab30455)
sheep anti-DIG (1:1500 Sigma-Aldrich, #11093274910)
Secondary antibodies used were raised in donkey and were conjugated with AlexaFluor 488, AlexaFluor 568, and AlexaFluor 647, all used at 1:1000; (Invitrogen, Carlsbad, CA):
Donkey anti-rabbit 488 #A-21206
Donkey anti-rat 488 #A-21208
Donkey anti-chicken 488 #A-21206
Donkey anti-rabbit 568 #A-10042
Donkey anti-goat 568 #A-21206

	Donkey anti-mouse 568 #A-10037<br>Donkey anti-rat 568 #A-78946<br>Donkey anti-rabbit 647 #A-31573<br>Donkey anti-sheep 647 #A-21448
Validation	All primary antibodies used in this study have either been described previously or have been valided using loss-of-function mutant animals

Eukaryotic cell lines

Policy information about cell lines

Cell line source(s)	COS cells used in this study were obtained from ATCC
Authentication	None of the cell lines used were authenticated
Mycoplasma contamination	Cell lines were not tested for mycoplasma contamination
Commonly misidentified lines<br>(See ICLAC register)	No commonly misidentified cell lines were used in the study

Animals and other organisms

Policy information about studies involving animals; ARRIVE guidelines recommended for reporting animal research

Laboratory animals	Both male and female mice have been used in this study. The age of mouse embryos and adult mice is specified in the method and the figure legends.<br>Mtg8-LacZ KI mouse (MGI:2183012)<br>Mtg16 mutant (MGI:3815201)<br>Lhx6 floxed mice (MGI:6466660)<br>Nkx2.1-Cre (MGI:3761164)<br>Lhx6-Cre (JAX 026555)<br>Nestin-Cre (JAX 003771)<br>Rosa26R-YFP (JAX 006148)<br>Rosa26R-tdTomato (JAX 007914)<br>Dlx1-lox-Venus-lox (MGI:4840325)
Wild animals	Not used in this study
Field-collected samples	No field collected samples were used in the study
Ethics oversight	United Kingdom Legislation (ASPA 1986)<br>European Union ethical standards outlined in the Council Directive 2010/63EU of the European Parliament

Note that full information on the approval of the study protocol must also be provided in the manuscript.
